# Polygenic Risk Score Predicts Modified Risk in *BRCA1* Pathogenic Variant c.4035del and c.5266dup Carriers in Breast Cancer Patients

**DOI:** 10.3390/cancers15112957

**Published:** 2023-05-28

**Authors:** Egija Berga-Švītiņa, Jeļena Maksimenko, Edvīns Miklaševičs, Krista Fischer, Baiba Vilne, Reedik Mägi

**Affiliations:** 1 Bioinformatics Lab, Rīga Stradiņš University, Dzirciema Street 16, LV-1007 Riga, Latvia; baiba.vilne@rsu.lv; 2 Institute of Oncology, Rīga Stradiņš University, Pilsoņu Street 13, Block 13, LV-1002 Riga, Latvia; jelena.maksimenko@rsu.lv (J.M.); edvins.miklasevics@rsu.lv (E.M.); 3 Pauls Stradiņš Clinical University Hospital, Pilsoņu Street 13, LV-1002 Riga, Latvia; 4 Department of Biology and Microbiology, Rīga Stradiņš University, LV-1007 Riga, Latvia; 5 Institute of Genomics, University of Tartu, Riia 23b, 51010 Tartu, Estonia; krista.fischer@ut.ee (K.F.); reedik.magi@ut.ee (R.M.); 6 Institute of Mathematics and Statistics, University of Tartu, Narva mnt 18, 51009 Tartu, Estonia

**Keywords:** polygenic risk score (PRS), breast cancer, ovarian cancer, *BRCA1* pathogenic variant carriers

## Abstract

**Simple Summary:**

The objective of our study was to explore the potential of using a polygenic risk score (PRS) to estimate the overall genetic risk of developing breast or ovarian cancer for women with inherited *BRCA1* pathogenic variants. We applied a previously developed PRS to 406 women with germline *BRCA1* pathogenic variants and found that the PRS accurately predicted breast cancer risk, but not ovarian cancer risk. These findings suggest that the use of the PRS may improve patient stratification and decision-making for breast cancer treatment and prevention strategies.

**Abstract:**

The aim of this study was to assess the power of the polygenic risk score (PRS) in estimating the overall genetic risk of women carrying germline *BRCA1* pathogenic variants (PVs) c.4035del or c.5266dup to develop breast (BC) or ovarian cancer (OC) due to additional genetic variations. In this study, PRSs previously developed from two joint models using summary statistics of age-at-onset (BayesW model) and case–control data (BayesRR-RC model) from a genome-wide association analysis (GWAS) were applied to 406 germline *BRCA1* PV (c.4035del or c.5266dup) carriers affected by BC or OC, compared with unaffected individuals. A binomial logistic regression model was used to assess the association of PRS with BC or OC development risk. We observed that the best-fitting BayesW PRS model effectively predicted the individual’s BC risk (OR = 1.37; 95% CI = 1.03–1.81, *p* = 0.02905 with AUC = 0.759). However, none of the applied PRS models was a good predictor of OC risk. The best-fitted PRS model (BayesW) contributed to assessing the risk of developing BC for germline *BRCA1* PV (c.4035del or c.5266dup) carriers and may facilitate more precise and timely patient stratification and decision-making to improve the current BC treatment or even prevention strategies.

## 1. Introduction

Breast cancer (BC) is the most common malignant tumor diagnosed among women in Western countries [1]. Every year, approximately 1200 and 300 women are diagnosed with BC and ovarian cancer (OC) in Latvia, respectively [2]. Female carriers of pathogenic variants (PVs) in high and moderate penetrance susceptibility genes, such as BRCA1 DNA repair-associated gene (*BRCA1*), BRCA2 DNA repair-associated gene (*BRCA2*), tumor protein p53 gene (*TP53*), partner and localizer of BRCA2 gene (*PALB2*), checkpoint kinase 2 gene (*CHEK2*), and ATM serine/threonine kinase gene (*ATM*), are at a highly increased risk of developing BC and OC compared with women in the general population [3,4]. Inherited PVs in the *BRCA1* gene are the most common cause of hereditary breast and ovarian cancer (HBOC). Associated lifetime risk (by the age of 80 years) for BC development has been evaluated in a variety of studies, with recent estimates ranging from 60% to 75% for female *BRCA1* germline PV carriers [5]. The corresponding OC risk has been estimated to be 34% to 44% for female *BRCA1* PV carriers [6,7]. In the Baltic region, previous research by several groups has demonstrated that two germline PVs in the *BRCA1* gene—c.4035del and c.5266dup—are founder variants in these populations and account for approximately 80% of all identified PVs in the *BRCA1* gene in BC and OC patients [4,8,9,10,11].

Currently, the clinical management of women carrying *BRCA1* PVs focuses on a combination of early diagnosis and cancer risk reduction through frequent screening, risk-reducing surgeries, and chemoprevention [12]. Preventive measures, such as bilateral mastectomy or risk-reducing salpingo-oophorectomy, are invasive and have substantial psychological and physiological effects. Accurate age-dependent estimates of cancer penetrance in *BRCA1* PV carriers are crucial in genetic counseling to make informed decisions about preventive measures that correspond to personalized BC or OC risk. Improved risk prediction may facilitate the identification of high-risk women who could benefit from early clinical intervention and low-risk women who may decide to delay prophylactic surgery or chemoprevention [5,12].

However, *BRCA1* PV-associated HBOC is inherited in autosomal dominant pattern with incomplete penetrance. Variable penetrance leads to challenging genetic counselling and risk assessment for each individual with *BRCA1* PV [13]. It has been demonstrated that for *BRCA1* PV carriers, the disease risk displays a polygenic pattern and mode of inheritance, with an elevated cancer risk observed in individuals with an increased number of affected first- and second-degree relatives. This observation suggests that other genetic factors modify cancer risk for *BRCA1* PV carriers [6,14,15,16]. Consistent with this observation, an increasing number of common BC and OC susceptibility single nucleotide variants (SNVs) have been identified through population-based genome-wide association studies (GWASs) that have demonstrated an effect on BC and OC risk in *BRCA1* PV carriers [6,17,18]. Although the identified SNVs confer small to modest risk individually, the combined multiplicative effect can be summarized using polygenic risk scores (PRSs) and incorporated into individual risk stratification [1,5]. PRSs are calculated by aggregating the weighted sum scores of risk alleles based on the effect sizes derived from GWAS results [19]. 

Global biobank initiatives and an increasing number of GWASs have created the opportunities to calculate more accurate effects of genetic markers, providing new tools for improved personalized risk assessments [14,20,21,22]. PRSs have the potential to be implemented in clinical risk models alongside independent biomarkers, such as *BRCA1* PV carrier status. Furthermore, many methods have been developed to compute PRSs, each with different strengths and weaknesses [20]. A common approach in PRS development involves using GWAS results to aggregate the effects of many genetic markers that are statistically associated with a specific trait or disease [19].

Recently, more complex PRSs with improved prediction accuracy have been developed using random-effects models. To develop joint PRS models containing a genome-wise set of 2,174,072 SNVs, the authors utilized a Bayesian grouped mixture of regressions model (GMRM), which jointly estimates genetic marker effects [23]. Two Bayesian approaches have been implemented for joint PRS model calculations: the age-at-onset BayesW and case–control BayesRR-RC model. The BayesW model was developed by Ojavee et al., providing probabilistic inference of the genetic architecture of age-at-onset phenotypes. It implements the parametrization of the Weibull distribution by using the logarithm (log) of the time-to-event and its moment, improving performance through enhanced variance estimation [24]. In simple terms, both models use a Bayesian approach, which makes probabilistic statements about or estimates the likelihood of outcomes, such as the status and age-of-onset of BC and/or OC, based on the input data, such as genetic factors. In our study, BayesW model was used to predict the age at which a woman may develop BC and/or OC based on her genetic information. For this, the BayesW model uses a specific type of probability distribution, the Weibull distribution, to model the time until the event, i.e., the onset of BC and/or OC. Moreover, the BayesW model uses a specific way of representing the Weibull distribution to model the age-at-onset of the disease. This involves taking the natural log of the time until the disease occurs (time-to-event) and using it along with a measure of the distribution’s shape (its moment) to define the parameters of the distribution. This approach allows for more accurate modelling of the age-at-onset of BC and/or OC and the genetic factors that contribute to it [24].

On the other hand, the grouped Dirac spike-and-slab model (termed BayesRR-RC), developed by Patxot et al., provides probabilistic inference of the genetic architecture by implementing an extended version of BayesR model. It provided estimates for group-specific variance, allowing for flexibility in prioritizing certain genomic regions (intronic, exonic, and distal regulatory regions) and demonstrating robust model performance [25]. In simple terms, our purpose of using the BayesRR-RC model was to understand the genetic factors that contribute to the onset of BC and/or OC. The model uses a statistical approach called a grouped Dirac spike-and-slab model to analyze the data. This approach enables the model to consider the fact that genetic markers can have different effects on different traits. The model is called a “spike-and-slab” model because it uses two different types of probability distributions to represent the effects of genetic markers on a trait or disease. The “spike” distribution represents markers that have no effect, while the “slab” distribution represents markers that do influence the onset of BC and/or OC. The model is also called a “grouped” model because it allows for the grouping of genetic markers into different categories based on their characteristics. For example, markers could be grouped based on their location in the genome (intronic, exonic, and distal regulatory regions) or their function. This approach allows us to identify which genetic markers are most likely to have an effect on a trait or disease and to estimate the size of that effect [25].

In this study, we investigated and compared the capacity of these two PRS models (BayesW vs. BayesRR-RC) to estimate the overall genetic risk of women carrying the 2 most common germline *BRCA1* PVs (c.4035del or c.5266dup) in the Latvian population to develop BC or OC due to additional genetic variations.

## 2. Materials and Methods

### 2.1. Study Cohort

Patients were selected based on two germline *BRCA1* PVs—NM_007294.4:c.4035del (rs80357711) and NM_007294.4:c.5266dup (rs80357906), regardless of family history of cancer. Both variants are frameshift variants that result in a premature stop codon and produce truncated (c.5266dup) or reduced (c.4035del) BRCA1 protein. The biological effect of the PVs is the loss of function of the protein. Based on the American College of Medical Genetics and Genomics (ACMG) classification, both variants are classified as pathogenic [26].

The study cohort consisted of 406 germline *BRCA1* PV (c.4035del or c.5266dup) carriers, recruited between 2002 and 2022, who were over 18 years at the recruitment. Among the cohort, 171 individuals were diagnosed with BC, 121 individuals were diagnosed with OC, and 114 individuals were unaffected. The diagnosis was confirmed through diagnostic germline testing at the Breast Surgery Unit of the Pauls Stradiņš Clinical University Hospital. All carriers had only one *BRCA1* PV (c.4035del or c.5266dup) present in a heterozygous state.

### 2.2. Genotyping with OncoArray

All the study samples were genotyped using the Infinium OncoArray-500K BeadChip (Illumina, San Diego, CA, USA) at the Institute of Oncology, Rīga Stradiņš University. The array comprises approximately 500,000 SNVs, including a genome-wide backbone of 250,000 tag SNVs representing common variants. The remaining markers encompass genetic variants associated with breast, ovarian, and other types of cancers, identified through GWAS and other approaches.

### 2.3. Genotype Calling and Quality Control (QC)

A standard genotype QC process was implemented for the dataset, which has been described in detail elsewhere [27]. Briefly, this process involved sample-based and SNV-based QC steps primarily using GenomeStudio software (Illumina, Genotyping module v2.0.5) and the command-line program PLINK v1.07 (Purcell et al., Boston, MA, USA) [28]. Samples with a call rate <98%, gender mismatch, race mismatch (>mean ± 6 SD) determined using principal component (PC) analysis [29], extreme heterozygosity (>mean ± 4.89 SD), inbreeding coefficient > 0.1, as well as SNVs located on chromosome Y, those with minor allele frequency (MAF) < 0.01, and those showing extreme deviation from Hardy–Weinberg equilibrium (HWE) (*p* < 1 × 10^−7^ for controls and *p* < 1 × 10^−12^ for cases) were excluded from the analysis.

### 2.4. Genotype Imputation

Missing genotypes for approximately 40 million SNVs were imputed for all individuals using the Estonian population-based high-coverage whole-genome sequencing (WGS) dataset (*n* = 2244) as the reference panel, as described previously [30]. A two-stage imputation approach was implemented, involving phasing with EAGLE v2.3 (Loh et al., Boston, MA, USA) [31] and imputation with BEAGLE v5.1 (Browning et al., Washington, WA, USA) [32]. Estimated genotypes were generated for approximately 38 million SNVs. Post-imputation QC was performed, excluding SNVs with MAF < 0.01 and imputation quality score DR2 < 0.8. The resulting filtered dataset consisted of 7,911,505 good-quality SNVs for subsequent analysis.

### 2.5. Polygenic Risk Score (PRS) Calculations

The PRS estimates employed in this study incorporated information from 2,174,072 SNVs that are present in both the UK Biobank (https://www.ukbiobank.ac.uk/ (accessed on 22 May 2023) [33]) and Estonian Biobank individuals (https://genomics.ut.ee/en/content/estonian-biobank (accessed on 22 May 2023) [30]). These PRSs were developed using data from 428,747 UK Biobank individuals and 105,000 Estonian Genome Center participants [23]. For the calculations conducted in this study, 2,041,044 SNVs were used due to the missingness of the remaining 133,028 variants in our dataset. The PLINK v2.00 function*-score* was used for all PRS calculations.

### 2.6. Statistical Analysis

For the statistical analysis, R v4.0.2 (R Core Team, Vienna, Austria) [34] and RStudio v1.3.1093 (RStudio Team, Boston, MA, USA) [35] software programs were used. All statistical tests conducted were two-sided, and *p* values below 0.05 were considered statistically significant. The association between PRS and the presence of BC and/or OC in *BRCA1* PV carriers was evaluated by using a binomial logistic regression model. The outcome variable had three categories: 0 (no cancer), 1 (BC), and/or 2 (OC). The model was adjusted for age, age squared, *BRCA1* PV (c.4035del or c.5266dup), and the first two PCs. Odds ratios (OR) and 95% confidence intervals (95% CI) were calculated using the R package Epi [36]. Receiver operating characteristic (ROC) curve analysis was performed to select the most optimal binomial logistic regression analysis model using the R package pROC [37].

## 3. Results

We studied 406 women who were carriers of one of *BRCA1* PVs with diagnosed BC or OC, or no cancer diagnosis at the time of recruitment. Among the study cohort, 171 women (42.1%) had been diagnosed with BC, and 121 women (29.8%) had been diagnosed with OC. The mean ages of disease onset were 46.67 years (range 25–92) and 50.55 years (range 27–79), respectively. The main characteristics of the study cohort are presented in Table 1.

Four different PRS joint models were employed for the risk calculations, i.e., score1–4. The description of each score is presented in Table 2.

We tested the association of four PRSs (score1–4) with the risk of BC or OC development using binomial logistic regression analysis, assessing, whether the two recently developed PRS models (BayesW vs. BayesRR-RC) are effective predictors of the BC or OC risk in *BRCA1* PV carriers in the Latvian population by comparing the PRS weighted effect size in mutation carriers with cancer (BC and/or OC) vs. in mutation carriers without cancer (controls).

**Figure 1:**
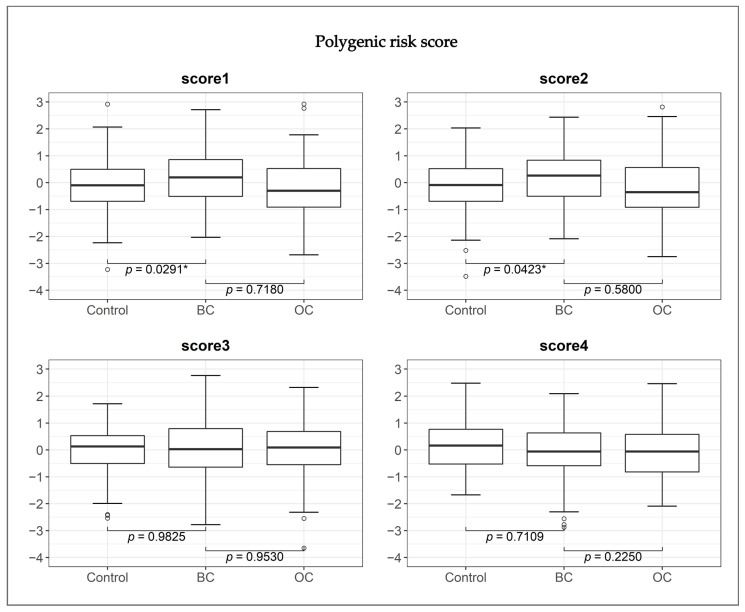
Boxplots and binomial logistic regression analysis *p* values of polygenic risk scores in 406 *BRCA1* PV carriers. Controls, no cancer; BC, breast cancer; OC, ovarian cancer. * *p* value below 0.05.

As a result, we observed that overall, the average PRSs (score1 and score2) calculated for BC patients were significantly higher in the BC group compared with the average PRS in the control group (Figure 1). The difference between the BC and control groups was statistically significant, with *p* values of 0.029 for score1 and 0.042 for score2. However, in the OC group, no statistical significance was observed (Figure 1, *p* > 0.05).

Among the four tested PRSs, score1 yielded the strongest association with the risk of developing BC (OR = 1.37; 95% CI = 1.03–1.81, *p* = 0.0291, Table 3). In addition to the PRS, we observed a significant association between the *BRCA1* PV c.5266dup and BC risk (OR = 2.55; 95% CI = 1.44–4.53, *p* = 0.0013). Regardless of the PRS employed, no statistically significant association was found between any of the PRSs and the risk of OC (Table 3). 

**Figure 2:**
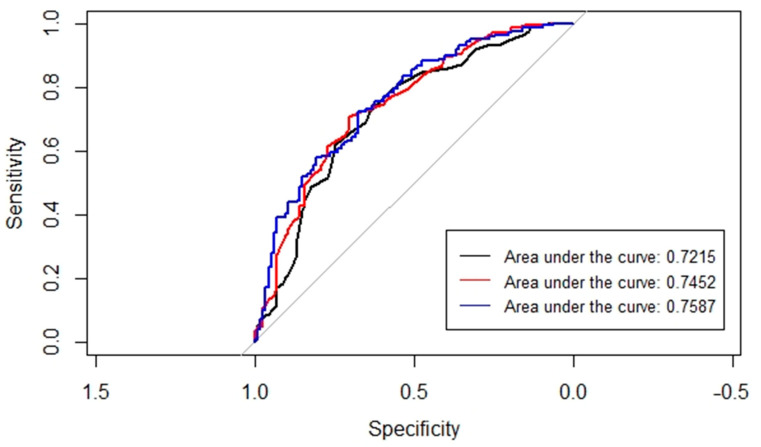
A comparison of the AUC (area under the receiver operating characteristic curve) to select the most optimal binomial logistic regression analysis model. In black—the model with only age and age squared as covariates; in red—the model with the *BRCA1* PV added; in blue—the model with the *BRCA1* PV and the best performing PRS added (i.e., score1).

The area under the receiver operating characteristic curve (AUC) analysis was performed to assess the predictive accuracy of three different models with varying covariates, including the PRS (Figure 2). The highest AUC value of 0.7587 was observed in the model that included age at onset, age squared, *BRCA1* PV, and the best-performing PRS (score1). Among the three models compared, our analysis using bootstrap method revealed a statistically significant difference (*p* value = 0.0368) specifically between the AUC of the model incorporating age and age squared as covariates and the model that incorporated age at onset, age squared, *BRCA1* PV, and the highest performing PRS represented by score1. 

## 4. Discussion

In this study, we investigated the association between two recently reported novel genome-wise PRSs [23], containing 2,174,072 SNVs, with the risk of BC and OC in *BRCA1* PV carriers. Although the best approach to select the SNV set and their weights to compute best performing PRS is still unknown, we hypothesized that by jointly estimating the effects of genome-wise SNVs in the PRS models, improved predictive performance could be achieved compared with commonly used approaches for PRS development [3]. Since most PRSs, as well as those evaluated in this study, are developed in population-based cohorts, it is urgent to carefully review and validate their performance specifically in *BRCA1* PV carriers to improve individualized risk assessments. The variable penetrance of germline PVs in the *BRCA1* gene presents challenges in estimating the likelihood, age, and site of disease onset in any individual, it is important to study how to schedule the initiation of screening and clinical management for high-risk women. PRS has the potential to stratify individuals based on their disease risk. To achieve this goal and implement PRSs in clinical practice, it is necessary to identify the most optimal set of SNVs that constitute the best-performing PRSs.

The results of the study demonstrate that the best-fitting BayesW PRS model could effectively predict the individual’s risk of developing BC, confirming the polygenic contribution to the development of BC phenotype in germline *BRCA1* PV carriers [6,12]. Although the BayesRR-RC PRS model performed well in predicting the risk of developing BC, the BayesW PRS model remained superior (Table 3).

Previously, only a few studies have focused on assessing PRS in individuals carrying PVs in the high-risk *BRCA1* gene. One notable study by Kuchenbaecker et al. [12] developed three PRSs for overall, estrogen receptor (ER)-positive and ER-negative BC, and one for OC patients. They utilized data from 15,252 female *BRCA1* PV carriers (BC = 7797, OC = 2462) and found strong associations between the PRS and BC and OC risk (particularly, the PRS for ER-negative BC displayed the strongest association with BC risk with a hazard ratio (HR)  =  1.27, 95% CI = 1.23–1.31, *p*  =  8.2 × 10^−53^). Another study in 9473 female *BRCA1* PV carriers with BC [6] demonstrated similar results, showing that the ER-negative PRS had the strongest association with BC risk for *BRCA1* PV carriers (HR = 1.29, 95% CI = 1.25–1.33, *p* = 3 × 10^−72^). ER-negative BC is the predominant tumor subtype in *BRCA1* PV carriers [38]; therefore, these studies highlight the strong association of BC subtype-specific PRS with the BC risk. This confirms that the best BC risk prediction accuracy can be achieved by implementing comprehensive clinical information in the analysis [6,12]. Unfortunately, in our study, the information concerning ER status could not be considered due to incomplete clinical data. The available information on ER status was only accessible for a small fraction (<80) of BC patients.

In the general population, studies have consistently demonstrated a strong association between PRS and overall BC risk (OR = 1.61, 95% CI = 1.57–1.65, with AUC = 0.630, 95% CI = 0.628–0.651) [1]. Consistent with previous studies, we observed that the calculated OR estimates for BC in *BRCA1* PV carriers are smaller than previously reported estimates in the general population, suggesting a potential subset of SNVs in PRS that do not combine multiplicatively with the *BRCA1* PV status. However, it is important to note that these results may not be directly comparable due to differences in sample size and study design [12].

Although previous studies have shown a strong association between PRS and OC risk (for example, Barnes et al. demonstrated that their developed high-grade serous PRS is strongly associated with OC risk (HR = 1.32, 95% CI = 1.25–1.40, *p* = 3 × 10^−22^) [6,12]), in our study no statistically significant association was observed. We observed that genome-wise PRS performed better in predicting BC risk than OC risk in *BRCA1* PV carriers (OR = 1.37, 95% CI = 1.03–1.81, *p* = 0.0291 * vs. OR = 0.99, 95% CI = 0.71–1.38, *p* = 0.9530). Our results might be explained by the relatively small number of 121 *BRCA1* PV carriers with the OC diagnosis in the study cohort.

The observation of a strong association between the *BRCA1* PV c.5266dup and BC risk can be explained by the mutation effect on the BRCA1 protein. It is well established that the *BRCA1* PV c.5266dup in exon 19 causes a frameshift and introduces a premature stop codon at position 74 of the new reading frame, which is located at the last exon. This mutant transcript is predicted to escape nonsense-mediated decay (NMD), and this variant is likely to yield a stable mutant truncated protein that lacks the C-terminal BRCT domain [39,40]. Similarly, *BRCA1* PV c.4035del in exon 10 causes a frameshift and introduces a premature stop codon at position 20 of the new reading frame. However, because all truncating mutations located in the exon 10 are subject to NMD, this mutation is also predicted to undergo NMD, resulting in a reduced protein yield [40]. Therefore, we can observe the genotype–phenotype correlation and differing clinical presentation based on the effect of *BRCA1* PVs on structural and functional changes in the mutated protein. It has previously been shown that the PVs located at the 3′ part of the *BRCA1* gene (e.g., c.5266dup) are associated with a higher risk of BC development, and PVs in exon 10 (e.g., c.4035del) exhibit almost equal BC and OC incidence among PV carriers [41]. In our data, the PV c.4035del did not provide statistically significant evidence for elevated BC risk compared with OC risk, supporting the observation that this *BRCA1* PV is associated with relatively equivalent risks of both cancers. It has been suggested that the *BRCA1* PV position could be an important additional variable in risk assessment [18].

There are several limitations in this research that should be noted, as they might have influenced the obtained results. In particular, the number of women with BC or OC who are carriers of germline *BRCA1* PV was relatively small in this study. Additionally, the study cohort does not reflect the general population of *BRCA1* PV carriers as the samples were obtained due to diagnostic germline variant testing in a clinical setting, which introduces potential selection biases. Although previous studies in our region have demonstrated that the tested *BRCA1* PVs—c.4035del and c.5266dup—account for approximately 80% of identified *BRCA1* PVs [4,8,9,10,11], it is important to acknowledge that this study focused only on these variants and did not investigate individuals with additional *BRCA1* PVs relevant to the development of BC and OC. This may result in an incomplete understanding of the genetic landscape and may not capture the full population of *BRCA1* PV carriers.

Second, we only analyzed 2,041,044 SNVs from 2,174,072 SNVs that were implemented in the PRS joint model (due to the missingness of the remaining 133,028 variants in our dataset). Different aspects, such as the quality of the DNA samples or microarray used, might have influenced the number of available SNVs. Additionally, one of the reasons for missing SNVs could be the imputation quality. Although we used a genetically similar reference panel provided by WGS data of 2244 Estonian biobank participants [30], certain genetic differences in the Latvian population remain that could potentially affect the PRS performance. Thus, the future improvement could be to increase imputation accuracy using a more population-specific reference panel from the Genome Database of Latvian Population (LGDB), when the respective WGS data will be obtained and available [42].

Third, as mentioned above, the lack of extensive clinical information on the particular tumor phenotypes was missing in a substantial proportion of our patients; therefore, our results represent average estimates of all phenotypes of BC or OC.

Finally, although the genome-wise PRSs used were developed in a population-based study, our results represent an independent evaluation of these PRSs in the *BRCA1* PV carriers of the Latvian population. We believe that genome-wise PRSs have the potential to be equally or even more predictive than previously developed PRSs. However, further validation with a larger study cohort of *BRCA1* PV carriers is needed, and our study can serve as preliminary data for a more comprehensive comparison of all available PRSs. Additionally, it is worth noting that our study design only considered the occurrence of a first BC or OC, and the risks of second or subsequent cancers were ignored. In future perspective, it would be valuable to investigate whether the tested PRS also contribute to the risk of developing secondary cancer in *BRCA1* PV carriers.

## 5. Conclusions

In conclusion, the PRSs tested in our study provide valuable information for assessing the risk of developing BC in germline *BRCA1* PV c.4035del and c.5266dup carriers. The data obtained in this study may have useful applications for risk assessment and when determining the appropriate age of implementation of BC prevention strategies in individuals with germline *BRCA1* PVs.

## Figures and Tables

**Table 1:** Patient characteristics. In all cases, the percentage of the total (N = 406) is given in brackets.

	Total	*BRCA1*:c.4035del	*BRCA1*:c.5266dup
**Study sample**	406	161 (39.66%)	245 (60.34%)
Breast cancer	171 (42.12%)	49 (12.07%)	122 (30.05%)
Ovarian cancer	121 (29.80%)	64 (15.76%)	57 (14.04%)
Controls	114 (28.08%)	48 (11.82%)	66 (16.26%)
**Mean age**	45.38	47.55	43.52
Breast cancer	46.67	49.53	45.52
Ovarian cancer	50.55	51.53	49.44
Controls	37.98	40.61	36.28

**Table 2:** Joint model characteristics employed for the risk calculations.

Score	Description
score1	The weighted effect size calculated in BC patients with the BayesW model
score2	The weighted effect size calculated in BC patients with the BayesRR-RC model
score3	The weighted effect size calculated in OC patients with the BayesW model
score4	The weighted effect size calculated in OC patients with the BayesRR-RC model

**Table 3:** Binomial logistic regression analysis results in three different study groups (BC, breast cancer; OC, ovarian cancer; BC + OC, both cancers combined). OR, odds ratios; 95% CI, 95% confidence interval for the associations of PRS with BC and/or OC risk in *BRCA1* PV carriers. Four different PRS joint models were employed for the risk calculations (see Table 2). * *p* value below 0.05; ** *p* value below 0.01.

	OR	95% CI	*p* Value
**BC + OC vs. Controls**			
score1	1.14	0.89–1.46	0.3119
score2	1.11	0.86–1.42	0.4205
score3	1.00	0.78–1.28	0.9781
score4	0.89	0.69–1.14	0.3514
*BRCA1*:c.5266dup	1.73	1.03–2.91	0.0375 *
**BC vs. Controls**			
score1	1.37	1.03–1.81	0.0291 *
score2	1.33	1.01–1.76	0.0423 *
score3	1.00	0.76–1.31	0.9825
score4	0.95	0.72–1.25	0.7109
*BRCA1*:c.5266dup	2.55	1.44–4.53	0.0013 **
**OC vs. Controls**			
score1	0.94	0.68–1.31	0.7180
score2	0.91	0.65–1.27	0.5800
score3	0.99	0.71–1.38	0.9530
score4	0.81	0.57–1.14	0.2250
*BRCA1*:c.5266dup	0.93	0.48–1.79	0.8170

## Data Availability

Summary statistics will be available from https://dataverse.rsu.lv/ (accessed on 22 May 2023) repository.
